# Neurovariability as a signature of adaptive brain function

**DOI:** 10.1007/s00429-026-03138-0

**Published:** 2026-06-12

**Authors:** Stephanie J. Forkel, Lilit Dulyan, Kurt Schilling, Michel Thiebaut de Schotten

**Affiliations:** 1 Donders Institute for Brain Cognition Behaviour, Radboud University, Nijmegen, the Netherlands; 2 Max Planck Institute for Psycholinguistics, Nijmegen, the Netherlands; 3 Department of Psychology, School of Human and Community Development, University of the Witwatersrand, Johannesburg, South Africa; 4 Brain Connectivity and Behaviour Laboratory, Bordeaux, France; 5 Vanderbilt University Institute of Imaging Science, Vanderbilt University Medical Center, Nashville, TN USA; 6 Groupe d’Imagerie Neurofonctionnelle, Institut Des Maladies Neurodégénératives-UMR 5293, CNRS, CEA, University of Bordeaux, Bordeaux, France

**Keywords:** White matter, Variability, Connectivity, Evolution, Personalised neuroscience

## Abstract

Neurovariability refers to the naturally occurring differences in brain structure and functional organisation across individuals. Rather than being noise, this variability reflects an adaptive feature of human neurobiology, supporting diversity, flexibility, learning, and resilience across evolution and lifespan. Here, we summarise the anatomical, functional, and evolutionary foundations of neurovariability, its relevance to health and disease, and the current limitations of methods for quantifying it. By understanding neurovariability as an emergent property of complex systems, we highlight its importance in advancing personalised neuroscience.

## What is neurovariability?

**Fig. 1:**
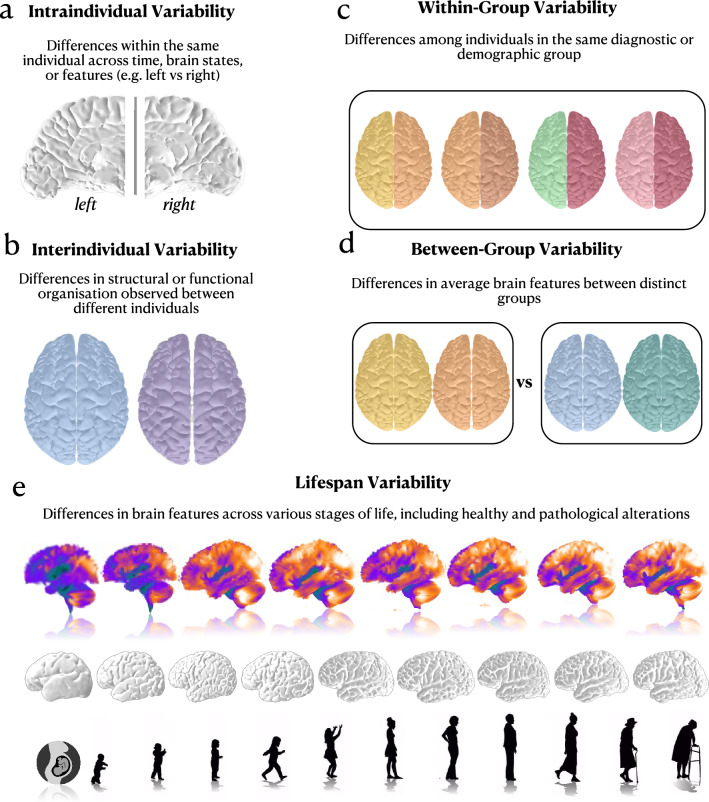
Schematic overview of different variability types. Intraindividual variability (**a**), interindividual variability (**b**), within-group variability (**c**), between-group variability (**d**), and across the lifespan (**e**) for the white matter (top, from birth until 75 + years) and grey matter (bottom, from 29 weeks of gestation to 70 years of age). The lifespan variability for the white matter is shown as deformation to a population template in mm, where warmer colours indicate more variability and colder colours more similarity between individuals (modified from Schilling et al. 2026). Data are available from the developing and young adult Human Connectome Project (www.humanconnectome.org)

Neurovariability refers to the natural, measurable differences in brain structure and function between individuals. These differences span from molecular anatomy (e.g., receptor distribution, Alves et al. 2025), cytoarchitecture (e.g., cell layering, Quabs et al. 2022; Amunts et al. 1999), surface anatomy (e.g., gyrification, Gregory et al. 2016), and connectional anatomy (e.g., white matter tracts, Thiebaut de Schotten, et al. 2011; Forkel et al. 2022) to functional signatures (e.g., neural fingerprinting, Finn et al. 2015). Historically dismissed as noise or biological imperfection, variability is increasingly recognised as a central, adaptive feature of brain organisation (Zilles and Amunts 2013; Bennett et al. 1964; Fig. 1).

Rather than being a source of error, neurovariability underpins cognitive, behavioural, and learning differences. From early developmental plasticity to preserved variability in ageing, this multi-layered scaffold supports the emergence of individual cognitive profiles.

## Historical, developmental, and evolutionary foundations

Early anatomists noted striking differences in the human brain’s folding patterns to the point where no two brains are completely identical in their gyral and sulcal patterns. This observation was already postulated in the nineteenth century by Heinrich Sachs as ‘the law of variability’ (Sachs 1892; Forkel et al. 2015). Variability is also observed in the arrangement and development of white matter anatomy. This principle was further reinforced by detailed post-mortem studies, which documented sulcal patterns across twenty-five hemispheres, revealing substantial interindividual differences in the shape, length, and positioning of sulci — particularly the secondary and tertiary folds (Ono 1990). This variability originates during development (Mihailov et al. 2025; Zilles et al. 2013), as tertiary sulci emerge later in gestation and even after birth, with their patterns shaped by genetic factors and structural constraints.

During early development, neurovariability is particularly pronounced. Synaptic overproduction, pruning, and environmental responsiveness produce a highly plastic brain organisation. This variability provides an exploratory space for adapting to individual contexts (Robinson et al. 2026; Knudsen 2004; Diamond 2001). Historically, the notion of sensitive periods is rooted in early behavioural work, which first demonstrated time-restricted windows for learning (e.g., imprinting, Lorenz 1935), face perception (Scott and Monesson 2009), speech perception (Werker and Tees 2005), and music perception (Hannon and Trehub 2005). This observation was later formalised at the neural level by Hubel and Wiesel’s work (Hubel and Wiesel 1970) on experience-dependent visual system development. These findings established that early variability is not merely noise but reflects a biologically constrained window for circuit tuning. Extending this framework to human cognition, it was proposed that language acquisition is similarly shaped by developmentally bounded periods of heightened plasticity (Lenneberg 1967), reinforcing the idea that early neurovariability plays a foundational role in structuring long-term functional organisation. For the brain’s white matter, similar relations between behaviour and anatomical variability have been shown. For example, literacy acquisition alters the white matter well into adulthood, but is especially potent within these developmental windows (Vanderauwera et al. 2018; Yeatman et al. 2014; Wandell and Yeatman 2013; Thiebaut de Schotten et al. 2014). In adulthood, neurovariability stabilises but remains critical with regard to functional vicariance—the capacity to perform the same cognitive task using different neural configurations. For example, the same memory task can be supported by medial temporal regions in younger adults, while older adults show increased prefrontal engagement (Deng et al. 2021). During healthy ageing, variability may increase as cognitive decline, compensatory mechanisms, and neural reorganisation unfold (Schilling et al. 2026; Bogdanov et al. 2025). Crucially, interindividual differences in developmental trajectories, alongside preserved capacity for flexible neural recruitment within individuals, have been linked to cognitive resilience, suggesting that a flexible neurobiological substrate supports adaptability in later life (Abrous et al. 2026).

Modern neuroanatomy, fuelled by neuroimaging methods, has revealed how extensive this variability is, cutting across all anatomical scales—from the molecular to the macroscale. Studies show that cytoarchitectonic fields vary in size and location across individuals (Amunts et al. 1999; Uylings et al. 2005), and receptor distribution exhibits pronounced interindividual differences (Palomero-Gallagher et al. 2019). Mesoscopic properties of the cortex, such as thickness, surface area, and volume, vary across individuals (Fischl and Dale 2000) and throughout the lifespan (Gogtay et al. 2004). At the macroscopic level, gyrification exhibits substantial individual differences (Fornito et al. 2004, 2006; Marie et al. 2015; Yousry et al. 1997; Galaburda et al. 1978; LeMay 1976). White matter pathways further demonstrate marked variability in morphology (Croxson et al. 2018), developmental and ageing trajectories (Lebel and Deoni 2018; Schilling, et al. 2023; Genon and Forkel 2022; Lebel et al. 2019; Kim et al. 2026), and hemispheric asymmetries (Thiebaut de Schotten et al. 2011; Forkel et al. 2014, 2022; Catani et al. 2007; Dulyan et al. 2025; Lilit Dulyan et al. 2026).

From an evolutionary standpoint, such variability is unlikely to be accidental. Croxson et al. (2018) suggest that interindividual variability is highest in association cortices, to support the adaptability of the brain's capacity for flexible and sophisticated information processing to ever-changing complex environments (Croxson et al. 2018). Zilles and Amunts (2013) emphasised that variability is not noise, but a foundational principle of brain organisation (Zilles and Amunts 2013). As Neil deGrasse Tyson remarked, “*the [variability in the] anatomy of the eye — and all of biology — makes no sense without evolution”* (Cosmos: A spacetime odyssey. 2014). This observation about the adaptive reshaping of the eye throughout evolution can be extended to neurobiology: the brain, like the eye, evolves incrementally through selection pressures that favour functional sufficiency and flexibility (Heuer et al. 2025).

## Neurovariability across scales

**Fig. 2:**
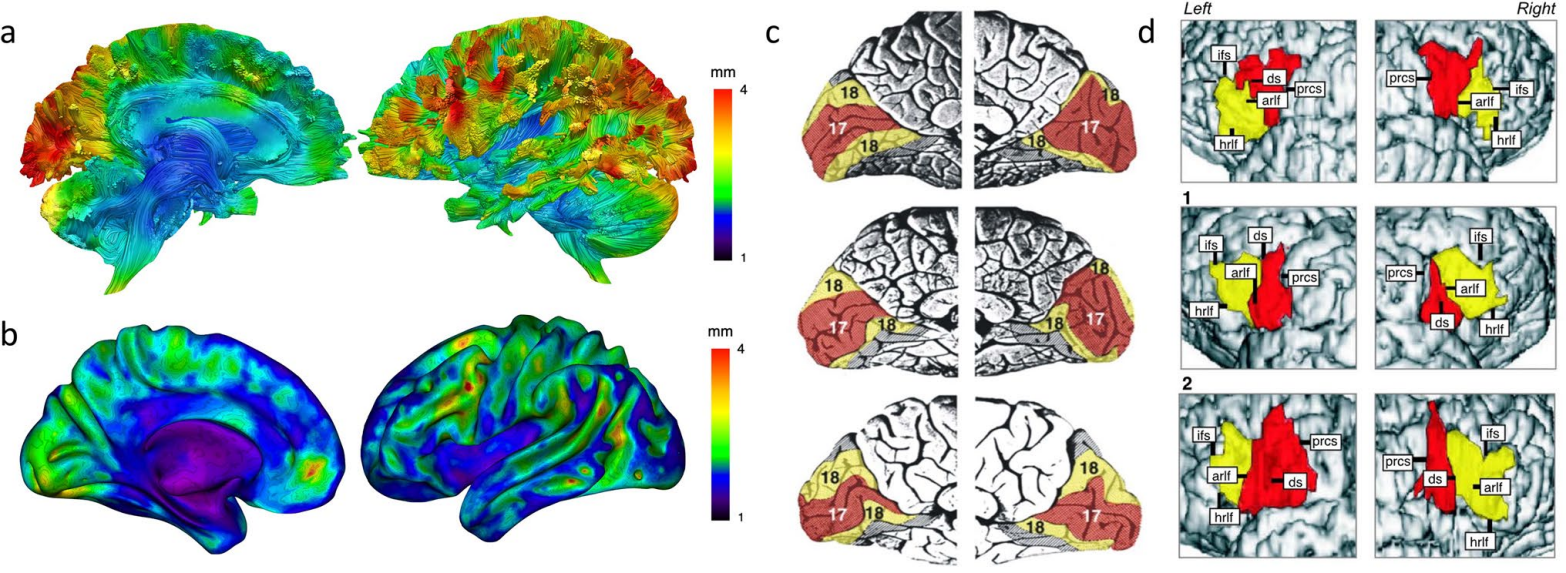
Variability across scales. Global variability at the white matter (**a**) and grey matter (**b**) level. Note: warmer colours indicate increased variability, reprinted with permission from Croxson et al. (2018). Cytoarchitectonic (**c**) and surface (**d**) variability at the intraindividual (left vs right hemisphere) and interindividual (between three specimens) level for visual (BA17/18) (Uylings et al. 2005) and language regions (BA44/45;Amunts et al. 2004).

Variability arises across multiple anatomical scales (Fig. 2): (1) microscale (e.g., receptor densities, gene expression), (2) mesoscale (e.g., cortical folding, white matter architecture), (3) macroscale (e.g., network configurations, hemispheric asymmetries).

## Variability in clinical populations

Neurovariability in clinical populations can be conceptualised along two complementary axes: variability within groups and variability between groups (see Glossary). Neurological and neurooncological conditions such as stroke, tumour, or traumatic brain injury are characterised by pronounced variability along both axes. Within groups, variability arises from heterogeneity in lesion location, extent, and network-level consequences. Between groups, this heterogeneity leads to broad dispersion in structural and functional profiles relative to healthy populations (Forkel et al. 2014, 2020; Hope et al. 2013; Saur 2006; Sharp et al. 2011; Duffau 2014; Derks et al. 2019; Fornito et al. 2015).

Psychiatric and neurodevelopmental conditions, including schizophrenia and autism, are likewise associated with substantial interindividual heterogeneity (Hahamy et al. 2015; Uddin et al. 2013), but this variability reflects divergent developmental trajectories rather than focal damage. As a result, individuals may exhibit distinct patterns of functional connectivity and network organisation despite shared diagnostic labels, consistent with transdiagnostic frameworks (Siugzdaite et al. 2020) that emphasise dimensional variation across, rather than within, traditional diagnostic boundaries.

Characterising and quantifying variability across these axes is critical for situating individual cases within population-level distributions and for distinguishing disorder-specific patterns from general principles of brain organisation (Bethlehem et al. 2022; Marquand et al. 2016). This dual-axis framework highlights that variability is not merely noise but a fundamental property of clinical populations, arising from different underlying mechanisms.

Importantly, this perspective encourages a reconceptualisation of clinical conditions not solely as deficits, but as shifts in the range or timing of neurobiological variability (Schilling et al. 2026, 2023).

## Current methods and limitations

Several methods aim to measure structural neurovariability. A commonly employed approach estimates the degree of deformation of an individual brain’s grey and white matter maps to a template (e.g., Croxson et al. 2018). These approaches offer the deformation in mm per voxel. These can be extracted as a variability map and projected onto the connectome or brain surface (Fig. 2a, b), highlighting areas or tracts of high and low variability. Another approach is normative modelling, which estimates a distribution of typical brain features (e.g., cortical thickness, white matter indices) and identifies individuals whose profiles deviate from the normative sample (Marquand et al. 2016). Brain charts across the lifespan are increasingly available for various global measures (Bethlehem et al. 2022; Ge et al. 2024) and specific structures (Lebel and Deoni 2018; Schilling et al. 2023; Kim et al. 2026). Despite these advances, current methods largely assume that neurovariability reflects deviations from a normative average and rely heavily on group-level representations. Moreover, such deviations, whether slight or extreme, do not necessarily distinguish atypicality from healthy developmental variation. Evidence from reading development illustrates this limitation: longitudinal work by Torppa and colleagues (2015) shows that children who initially fall below diagnostic thresholds may later outgrow their diagnosis, while others not identified early develop late-emerging dyslexia (Torppa et al. 2015). This suggests that early deviations from normative benchmarks may, in some cases, reflect transient developmental variation rather than persistent disorder, while stable difficulties may not always be detectable at early stages as they fall above the percentile cutoff. The dominance of research in Western college populations further narrows the scope of observed variability, likely overlooking significant anatomical and functional diversity within and across populations (e.g., Isamah et al. 2010; Tang et al. 2018; Rao et al. 2017). These limitations highlight a fundamental gap: current structural approaches primarily capture variability as deviation from a static reference, but do not account for how brain organisation unfolds dynamically over time or how individuals may implement different neural configurations to achieve similar functions. To address this, complementary approaches have emerged that characterise neurovariability in the structural (Schilling et al. 2026) and functional domains (Finn et al. 2015; Hutchison et al. 2013), focusing on individual specificity of brain network organisation. A common feature that is, however, missing across all these methods is the absence of temporal dynamics.

An additional consideration is the role of test–retest reliability and measurement variability in shaping estimates of neurovariability. Without accounting for these factors, there is a risk that noise may be misinterpreted as meaningful individual variation (Zilles and Amunts 2013). Structural and diffusion MRI measures, for example, are influenced by scanner differences, acquisition protocols, preprocessing pipelines, and within-subject variability across sessions. Empirical work has shown that variability in diffusion MRI metrics can arise from both biological and methodological sources, underscoring the need to carefully disentangle these contributions (e.g., Schilling et al. 2021; Dell’Acqua et al. 2025). Although methodological advances continue to improve reliability, estimates of variability remain constrained by measurement precision. This highlights the importance of distinguishing true biological variability from methodological noise, for instance, through repeated measurements, explicit modelling of reliability, and harmonisation approaches. Capturing the full scope of human neurovariability will require expanding these methods to more diverse populations and combining them with complementary approaches that assess variability at different anatomical and temporal scales.

## Theoretical advances

Neurovariability can be understood as an emergent property of complex systems, arising not from fixed group-level traits but from dynamic interactions among genetic, environmental, and developmental influences. One key principle is functional vicariance—the brain’s capacity to achieve the same behavioural outcome through different neural routes—which underpins cognitive flexibility and resilience. As such, variability is not a biological error but a lawful consequence of experience operating within fixed anatomical scaffolds. In addition, variability appears to operate within an adaptive window, where too little or too much may signal pathology. For example, neurological and psychiatric conditions are often associated with increased differences in brain structure and function relative to healthy populations, which may reflect shifts in the magnitude or distribution of neurovariability. Pathology, as such, is a reconfiguration of the variability landscape that alters brain function and adaptability. A key theoretical challenge lies in defining the range and boundaries of typical neurovariability: what constitutes the expected spectrum of individual differences, at what point deviations reflect atypicality or pathology rather than adaptive diversity, and what tools can detect and quantify such departures (Chamberland et al. 2021; Di Biase et al. 2023).

## Conclusion

Neurovariability is an adaptive feature of brain architecture, honed by evolution to support learning, flexibility, and resilience. It manifests across scales and the lifespan and is central to cognition, development, and functional recovery. Mapping and modelling neurovariability and recognising its complexity across different contexts and populations is essential for advancing personalised neuroscience. By embracing the diversity of human neural organisation, we move closer to understanding individuality and universality in brain function.

## Data Availability

No datasets were generated or analysed during the current study.

## Glossary of variability terms

Intraindividual variability: Differences within the same individual, either across time (e.g., days), across conditions (e.g., task states or brain states), or across features (e.g., hemispheric asymmetry, see Figure 1a).
Interindividual variability: Refers to differences between individuals in general. This broadest category refers to studies that examine how interindividual differences can reveal insights into cognitive processes, brain-behaviour relationships, and structural-functional correlations (see Figure 1b).
Within-group variability: A subset of interindividual variability, specifically among people within the same defined group (e.g., age group, diagnostic category). It reflects how much individuals within a group differ from each other. High within-group variability can mask group-level effects, especially in case-control designs (see Figure 1c).
Between-group variability: Refers to differences in group means, often used to compare clinical vs. neurotypical populations, or different age cohorts. Traditional statistical measures (e.g., ANOVA, t-tests) are commonly used to determine these group differences. However, between-group effects can be misleading if within-group variability is high (see Figure 1d).
Lifespan variability: Changes in brain structure and functional organisation observed across different stages of life — from high developmental plasticity in childhood to stability in adulthood, and adaptive or pathological changes in ageing (see Figure 1e).
Neurovariability: A form of interindividual variability that refers to the natural, measurable differences in brain structure and function between individuals (i.e. how different one brain is from another).
